# Optimising a multi-strategy implementation intervention to improve the delivery of a school physical activity policy at scale: findings from a randomised noninferiority trial

**DOI:** 10.1186/s12966-022-01345-6

**Published:** 2022-08-20

**Authors:** Cassandra Lane, Luke Wolfenden, Alix Hall, Rachel Sutherland, Patti-Jean Naylor, Chris Oldmeadow, Lucy Leigh, Adam Shoesmith, Adrian Bauman, Nicole McCarthy, Nicole Nathan

**Affiliations:** 1 School of Medicine and Public Health, The University of Newcastle, Newcastle NSW, 1 University Drive, Callaghan, NSW 2308 Australia; 2 Hunter New England Population Health, Hunter New England Area Health Service, Newcastle, NSW Australia; 3 Priority Research Centre for Health Behaviour, The University of Newcastle, Newcastle, NSW Australia; 4 Hunter Medical Research Institute, New Lambton Heights, NSW Australia; 5 School of Exercise Science, Physical and Health Education, University of Victoria, Victoria, BC Canada; 6 School of Public Health, University of Sydney, Sydney, New South Wales Australia

**Keywords:** physical activity, policy, implementation, optimisation, adaptations, school, children, noninferiority, scale-up

## Abstract

**Background:**

To maximise their potential health benefits, school-based physical activity policies need to be implemented at scale. This paper describes the third in a sequence of trials that sought to optimise an effective strategy (PACE) to assist schools’ implementation of a physical activity policy. Specifically, it aimed to determine the probability that a multi-strategy intervention adapted to reduce in-person contact (Adapted PACE) was “as good as” the original intervention (PACE) in increasing the weekly minutes of structured physical activity implemented by classroom teachers.

**Methods:**

A noninferiority cluster randomised controlled trial was undertaken with 48 primary schools in New South Wales, Australia. Schools were randomised to receive PACE or a model with adaptations made to the delivery modes (Adapted PACE). Teachers’ scheduled minutes of weekly physical activity was assessed at baseline (Oct 2018-Feb 2019) and 12-month follow-up (Oct-Dec 2019). The noninferiority margin was set at − 16.4 minutes based on previous data and decision panel consensus. A linear mixed model analysed within a Bayesian framework was used to explore noninferiority between the two PACE models. A cost minimisation analysis was conducted from the health service provider perspective, using the Australian dollar (AUD).

**Results:**

The posterior estimate for the between group difference at follow-up was -7.48 minutes (95% credible interval=-19.18, 4.10 minutes). There was an estimated 93.4% probability of Adapted PACE being considered noninferior (only 6.6% of the posterior samples crossed the noninferiority margin of -16.4 minutes). That is, the minutes of physical activity implemented by teachers at Adapted PACE schools was not meaningfully less than the minutes of physical activity implemented by teachers at PACE schools. The mean total cost was AUD$25,375 (95% uncertainty interval = $21,499, $29,106) for PACE and AUD$16,421 (95% uncertainty interval = $13,974, $19,656) for Adapted PACE; an estimated reduction of AUD$373 (95% uncertainty interval = $173, $560) per school.

**Conclusions:**

It is highly probable that Adapted PACE is noninferior to the original model. It is a cost-efficient alternative also likely to be a more suitable approach to supporting large scale implementation of school physical activity policies.

**Trial registration:**

Retrospectively registered with the Australian New Zealand Clinical Trials Registry (ACTRN12619001229167).

**Supplementary Information:**

The online version contains supplementary material available at 10.1186/s12966-022-01345-6.

## Background

To achieve the World Health Organization’s (WHO) goal of a 15% relative reduction in the global prevalence of physical inactivity by 2030, they have recommended the implementation of school physical activity policies [1]. Accordingly, a number of countries including Australia [2], Canada [3], the United States (U.S.) [4], Denmark [5], China [6], and England [7] have policies mandating a minimum time that schools are to provide students with structured physical activity across the school week [2–4, 8]. Despite this, school physical activity policies are often poorly implemented [3, 4, 8–13]. For example, studies in Canadian elementary schools found less than half of teachers were implementing the mandatory provincial physical activity policy [14, 15], and an Australian study found that only 30% of teachers were scheduling the state-required 150 minutes of physical activity across the school week [16].

To achieve population-wide implementation, and maximise the public health benefits of school physical activity policies, support to assist schools in overcoming barriers to their implementation is required [17]. Comprehensive multi-strategy interventions have been found effective in improving school’s physical activity policy implementation. For example, recent randomised controlled trials (RCTs) in Australia tested strategies which have largely relied on in-person teacher training, technical assistance, educational outreach, and ongoing support [16, 18]. These strategies substantially improved policy implementation [16, 18] however they were resource-intensive, requiring significant investment (absolute cost) and workforce infrastructure to deliver [19]. While effective, such approaches may be cost prohibitive and unsuitable for use by government health and education agencies responsible for supporting large scale implementation of physical activity policies [20].

Optimisation is an emerging innovative concept in public health that may offer a means of improving the impact of physical activity policy implementation strategies by enhancing their capacity to be delivered at scale [21]. Optimisation is a data driven process that seeks to maximise the impact of implementation strategies, within resource constraints, through repeated testing and strategy refinement [21]. In the case of school-based physical activity policies, optimisation processes could be used to improve population-level impacts through adapting implementation strategies in a manner that they achieve similar effects but can be delivered on a greater scale. For example, via adaptations to the modality of delivery of implementation strategies [20] such that more expensive (e.g., in-person) modes of support are replaced with modes that afford greater reach at lower costs [22].

While we are not aware of optimisation processes being applied to any school physical activity policy or program implementation research, the approach has previously demonstrated potential merit in this setting [23, 24]. For example, the incremental cost-effectiveness ratio of a multi-strategy intervention to assist schools’ implementation of a nutrition policy were substantially reduced over a series of sequential RCTs by removing strategies or adapting their mode of delivery [23]. The ‘optimised’ implementation intervention was then adopted as part of a jurisdictional wide scale-up by health services in Australia [23, 24]. Given the potential benefits, we conducted a sequence of trials to optimise our multi-strategy intervention which supports schools’ implementation of a mandatory physical activity policy (Physically Active Children in Education [PACE] [16, 18, 25]) for delivery at scale. This paper describes the third in that sequence of trials, that aimed to determine if a multi-strategy intervention with mode of delivery adaptations (to reduce in-person contact; Adapted PACE), was “as good as” [26] (noninferior) the original intervention (which relied on more in-person delivery of implementation strategies; PACE), in increasing the weekly minutes of structured physical activity implemented by classroom teachers. We also explored whether adapting the more expensive modes of delivery maintained a meaningful effect but at a lower cost.

## Methods

This study is reported in accordance with the CONSORT statements for noninferiority and equivalence randomised trials [27], cluster RCT [28], and the Standards for Reporting Implementation Studies (StaRI) statement [29]. Ethics approval was obtained from the Hunter New England Human Research Ethics Committee (2019/ETH12353), The University of Newcastle Human Research Ethics Committee (H-2008-0343), as well as the New South Wales (NSW) Department of Education (SERAP no.2017184) and the relevant Catholic School Offices. Assay sensitivity (i.e., plausible reason to believe that the comparator would be more effective than no intervention [30–32]) was ascertained through emulating the conditions (e.g., school type, participants, study region, delivery personnel and processes) of previous trials that established efficacy of PACE [16, 18, 25].

### Study design and setting

A two-arm, cluster randomised controlled noninferiority trial was conducted in 48 schools from the Central Coast (CC; *n* = 8) and Hunter New England (HNE; *n* = 40) regions of NSW. Collectively these regions have approximately 572 primary schools [33] across a geographic area of more than 130,500 km^2^. They consist of a socioeconomically and demographically diverse population of approximately 158,000 children aged 5–14 years [34].

### Participants and recruitment

The sampling pool for this trial consisted of all government, independent and Catholic schools in the study region, excluding those that had participated (past or current) in another physical activity intervention (including a previous PACE trial), or catered exclusively for children with special needs. A study information package was emailed to school principals and those interested were asked to provide written informed consent. Teachers at consenting schools were provided with a brief overview of the study purpose, invited to participate, and informed that completion of an evaluation survey represented their consent to participate.

### Randomisation and blinding

Following baseline data collection, an independent statistician used a computerised random number function to randomise schools (1:1) to receive the original PACE or the Adapted PACE multi-strategy intervention. Allocation was stratified by region (CC and HNE) and geographic (rural versus urban) location of each school given evidence of an association between these factors and the trial outcome [3]. Schools were not informed of their allocation. At follow-up, data collectors were not blind to group allocation.

### Multi-strategy implementation interventions

#### Group 1 – PACE

PACE was designed to assist school’s implementation of the NSW Department of Education sport and physical activity policy which requires schools to schedule 150 minutes of physical activity across the school week [35]. This may include time in: physical education (PE), sport, or other structured activities such as energisers (3–5 minute classroom physical activity breaks) and active lessons (physical activity integrated into literacy lessons) [36]. Table 1 outlines the eight original PACE implementation strategies. PACE was designed using the Behaviour Change Wheel (BCW) [37] and Theoretical Domains Framework (TDF) [38], with strategies purposefully selected to overcome school-level barriers to the scheduling of physical activity. Barriers were identified following extensive formative research which included (i) literature reviews; (ii) interviews with primary school teachers and (iii) observations of teachers’ delivery of PE, sport and the school environment. Implementation strategies were selected following the recommended process described by Michie et al. [37], mapping identified barriers to the BCW and TDF. An advisory group consisting of implementation and health behaviour scientists, physical activity experts, teachers, principals and senior government policy makers then assessed each strategy for its Affordability, Practicality, Effectiveness and cost-effectiveness, Acceptability, Side-effects/safety and Equity (APEASE). This helped reduce the number of strategies and or make them relevant to the context. A complete description of intervention development is published elsewhere [18, 25].

**Table 1:** Description of PACE implementation strategies, including a comparison between PACE and Adapted PACE

Implementation strategy and barrier(s) addressed	Link to the BCW (*TDF domain*)^a^	Implementation strategy description	PACE		Adapt-ation	Adapted PACE	
			Mode of delivery	Delivery provider		Mode of delivery	Delivery provider
1. **Centralise technical assistance and provide ongoing consultation** with ≥1 expert in the strategies used to support implementing the innovation Barriers: Teachers knowledge, ability or competence; Lack of time; Perceived priority of the policy in the schools	Psychological capability (*beliefs about capabilities; knowledge*) Opportunity: social (*environmental context and resources*) Motivation: reflective (*goals*)	Project officers (a PE teacher and health promotion practitioner) employed by the health service provided technical assistance to in-school champions throughout the study period (12 months). Their role was to provide in-school champions with expertise, advice and resources to help them problem solve barriers to policy implementation.	In-person (at the school) & email/ telephone	Project officer	✓ →	Email/ telephone	Project officer
2. **Mandate change** Barriers: Support from school boards; Physical activity considered a lower priority than other subjects	Opportunity: social (*social influences*) Motivation: reflective (*goals*)	**2a.** Project officers had one ×1-hr meeting with school principals and school executives to communicate the importance and benefits of the policy and gain their commitment for policy implementation over the school year.	In-person (at the school)	Project officer	✓ →	Email/ telephone	Project officer
		**2b.** School executives were asked to demonstrate their commitment to implementing the policy through the development of a school policy (‘Sport and Physical Activity Procedures document’) as required by the policy.	N/A	N/A	×	N/A	N/A
		**2c.** Principals and school executives were asked to demonstrate their support for, and the importance of, the policy by communicating to the broader school community (e.g., via newsletters, assemblies and staff meetings) that the implementation of the policy was a school priority and expected of all staff.	In-person (at the school) & email/telephone	Principal/ school executive	×	In-person (at the school) & email/telephone	Principal/school executive
3. **Identify and prepare champions** Barriers: Lack of time in the curriculum; Teachers knowledge, ability or competence	Opportunity: social (*environmental context and resources*) Psychological and physical capability (*beliefs about capabilities*)	**3a.** Each school nominated one to two in-school champions (existing teacher(s) at the school) who, with the support of the project officer, were responsible for leading the development and implementation of the policy in their school over the a 12 month period.	N/A	N/A	×	N/A	N/A
		**3b.** In-school champions attended one × full day (5-hour) training workshop with in-school champions from other schools (max 20 participants). These workshops covered: education about the policy, instruction and demonstration of energisers, active lessons and PE lessons, identification of barriers/ facilitators to implementation and possible solutions to overcome these. They also provided time to begin action planning. The training was accredited by the state educational authority and provided teachers continuing professional development hours.	In-person (at venues with conference and catering facilities)	Project officer	×	In-person (at venues with conference and catering facilities)	Project officer
4. **Develop a formal implementation blueprint** Barrier: Perceived priority of the policy in the schools	Motivation—reflective (*goals*)	In-school champions were supported to develop a plan for policy implementation in their school. The plan identified what the school was aiming to achieve, the strategies to do so and by when, and the resources available or required. The plan was segmented into school terms to break up the more complex policy requirements into achievable tasks.	N/A	N/A	×	N/A	N/A
5. **Conduct educational outreach visits** Barrier: Teachers knowledge, ability or competence	Psychological and physical capability (*beliefs about capabilities*)	School staff attended one × 1 to 2-hour information and training session delivered during a school staff meeting. Teachers were provided with an overview of the policy, including its importance and requirements for implementation. The plan for their schools’ implementation of the policy was presented as well as the timeline of expected key milestones. Staff participated in practical demonstrations of suggested physical activities (e.g., energisers and active lessons) which they could incorporate into their normal classroom routines.	In-person (at the school in staff meeting room)	Project officer	✓ →	In-person (at the school in staff meeting room)	In-school champion
6. **Develop and distribute educational materials** Barrier: Teachers knowledge, ability or competence	Psychological capability (*beliefs about capabilities; knowledge*)	**6a.** In-school champions received an ‘intervention manual’ inclusive of policy templates as well as examples of a physical activity timetable and PE curriculum.	Intervention manual	N/A	×	Intervention manual	N/A
		**6b.** In-school champions and teachers received educational materials during their respective training sessions. These resources included ideas and strategies for practical games for increasing physical activity during class time, example timetables etc.	Print copies and online portal	N/A	×	Print copies and online portal	N/A
		**6c.** In-school champions and teachers were provided access to professional learning videos which reinforced information received during their respective training sessions. In-school champions were asked to view the videos and to organise a time for their colleagues to watch them during a staff meeting.	Online Portal	N/A	×	Online portal	N/A
7. **Capture and share local knowledge** Barriers: Teachers knowledge, ability or competence; Lack of time in the curriculum	Opportunity: social (*social influences*) Motivation: reflective (*belief about consequences*)	In-school champions and teachers were provided access to case studies from other schools. Case studies described ‘success stories’ of how in-school champions and teachers had overcome frequently reported barriers to implement the policy in their school.	Online Portal	N/A	×	Online portal	N/A
8. **Change physical structure and equipment** Barrier: Availability of equipment	Opportunity: physical (*environmental context and resources*)	**8a.** Each school was provided with a physical activity pack consisting of items such as bean bags, balls, hoops etc., to engage in activities advertised in educational materials and/or exemplified during the in-school champion workshop and teacher training.	N/A	N/A	×	N/A	N/A
		**8b.** In-school champions were encouraged to develop classroom physical activity packs for all teachers using existing school sport equipment. These packs were to be kept in each classroom enabling teachers to implement suggested activities more easily.	N/A	N/A	×	N/A	N/A

^a^Please see Nathan et al. [25] for a more detailed explanation of the hypothesised mechanisms of action via the BCW and TDF

#### Group 2 – ‘Adapted PACE’

The multi-strategy PACE intervention was adapted for delivery at scale by the local health service [39]. No PACE strategy was considered discretionary (non-core) as each was theoretically derived and evidence-informed to address specific barriers of the target behaviour. Drawing on evidence from studies of scaled-up health interventions in schools [20, 23, 40–42], it was hypothesised that adaptation of implementation delivery would not substantively reduce effects. In doing so, this would reduce costs to the health service to deliver the implementation support. Table 1 includes an overview of the mode of delivery adaptations that were made to several PACE implementation strategies (1a, 3 and 5) following a rigorous decision making process (Additional file 1). Additional file 2 provides a report of the adaptations in accordance with the Framework for Reporting Adaptations and Modifications to Evidence-based Implementation Strategies (FRAME-IS) [43]. The final Adapted PACE met the needs of stakeholders and fit within resource constraints of the local health service. Briefly, strategy 1 (centralised technical assistance and ongoing support) and strategy 2a (mandated support via engagement of school principal) delivered via email/telephone rather than in-person, and strategy 5 (educational outreach) delivered by an in-school champion rather than an external project officer.

### Data Collection

#### Primary outcome: weekly minutes of structured physical activity implemented by classroom teachers

Robust noninferiority testing requires use of the same outcome measure that was used in a previous trial in which the comparator was proven effective. Consistent with the previous PACE trials [16, 18], teacher’s mean minutes of scheduled physical activity (total of PE, sport, energisers and/or active lessons) was measured using a daily log-book completed during a one-week period at baseline (October 2018–February 2019) and 12-month follow-up (October–December 2019). Data from log books were considered valid and included for analysis if teachers had recorded no more than 250 minutes of physical activity across the full five-day school week.

#### Secondary outcomes: weekly minutes of energisers, active lessons and PE implemented by classroom teachers

Secondary outcomes included the mean weekly minutes scheduled by teachers for each of PE, energisers and active lessons in the daily activity logbook. Sport was excluded as a secondary outcome as no significant effect has been established for sport in previous trials [16, 18]*.*

#### Program delivery costs

Program implementation costs were calculated from the health service provider perspective in Australian dollars (AUD) using 2019 as the base-year value (AUD$1 = approximately $0.69 U.S. dollars). Costs incurred for research or program development were excluded as they were not associated with any difference between groups. Cost-related activities were extracted from project officer records, coded by strategy, and transferred into an economic spreadsheet. Relevant costs included (i) project officer salaries; (ii) in-school champion training workshop expenses such as venue hire (actual rates charged), catering and staff reimbursement to attend; and (iii) the development and distribution of PACE resources such as physical activity equipment packs and in-school champion manuals. Consumable costs were measured directly using project records.

#### School and participant characteristics

Details of each schools’ type, size (number of students), relative socio-economic position, and geolocation at baseline were retrieved online via the Australian Curriculum, Assessment and Reporting Authority (ACARA) [44]. Surveys of school principals and classroom teachers were used to collect demographic information (sex, age, employment status, years teaching, and if they were a specialist PE teacher) as well as details regarding the operational characteristics of schools, school participation in other physical activity programs, and PACE implementation activity. Any differences in baseline characteristics of those participants who completed primary outcome data and those who dropped out from the study were investigated. Items were sourced from previous surveys of school principals conducted by the research team which have achieved participation rates of between 70 and 96% [45].

### Analysis

#### Noninferiority analysis

An intention to treat approach was applied, with all available valid data included in all analyses. Mixed models within a Bayesian framework were used to compare, between PACE and Adapted PACE, the minutes of implemented weekly physical activity in terms of total physical activity (primary outcome) and PE, energisers and active lessons (secondary outcomes). All models included a random level intercept for school, to account for clustering, and a fixed effect for experimental group. A linear mixed model was employed for the primary outcome, with a fixed effect included for the baseline value of the outcome. Missing baseline and follow-up data were imputed within this model, where missing outcome values were drawn from their posterior predictive distribution using the one-step approach [46]. Gamma-hurdle models were used to analyse the secondary outcomes, as a linear distribution did not fit these outcomes. Each of these models included a gamma distribution with a log link function to model the non-zero values, and a logit link function to model the zero values of the outcome. A longitudinal model rather than a baseline adjusted model was employed due to difficulties with model convergence when attempting to impute missing data. Therefore, the models for the secondary outcomes also included a fixed effect for time and a time-by-experimental group interaction term. All models applied an uninformative prior distribution, and were then replicated using an informative prior distribution based on knowledge of the distribution of the PACE strategy obtained from the previous effectiveness trial [16]. The No-U-Turn sampler (NUTS) was used to obtain estimates from the posterior distribution for all parameters, with a burn in period of 10,000, 10,000 post-burn in samples, and 4 chains. Convergence was assessed by inspecting the trace plots of the parameters, the reported effective sample size, and the Gelman-Rubin statistic. The mean posterior estimate and 95% credible intervals are reported for all outcomes. Furthermore, the posterior probability distribution was used to determine the probability that adapted PACE was noninferior to the original model for the primary and secondary outcomes, relative to their pre-specified noninferiority margin (see below). A variance ratio, which is the ratio of the variance of the posterior predictive distributions not conditioning on group level terms, to the variance of the posterior predictive distribution conditioning on all random effects (a Bayesian equivalent to ICC), was calculated for each model [47].

#### Sample size and noninferiority margin

This trial had a fixed sample size of 48 schools. The noninferiority margin (∆) for the primary trial outcome was set at − 16.4 minutes of scheduled physical activity. The ∆ is an “acceptable” between-group difference based on previous trials of the reference treatment combined with clinical judgement [48]. The ∆ for the current study was informed by usual practice for determining noninferiority margins in clinical trials (often set to maintain 50% of the lower confidence interval from a trial in which the comparator was proven effective) [30], as well as input from an expert decision panel as to what value would be considered beneficial. It was calculated as per recommendations [30, 32, 48]:

**Table:** 

∆ = (1-‘acceptable proportion of the effect size retained’) × (lower bound confidence interval) ∆ = (1–0.50) × 32.8 ∆ = 16.4* *A favourable result in the outcome is expressed as an increase in minutes therefore, our noninferiority margin is set as − 16.4 minutes, meaning that for Adapted PACE to be deemed noninferior it should not be any lower than 16.4 minutes than PACE.	

The ∆ decision panel, consisting of the PACE research team and stakeholders, implementation scientists, and experts in schools and physical activity, deemed that 50% was an acceptable proportion of the effect size retained. Panel discourse was informed by: reviews of similar studies with comparable outcomes [40, 49–51]; quantified attenuation in effect sizes associated with physical activity interventions adapted for scale-up [20]; evidence of the inverse dose-response relationship between physical activity and health outcomes [52], supporting an ‘anything is better than nothing’ notion [53] and; with consideration that full policy implementation may occur overtime, and in a nonlinear manner, as practices normalise within schools [54, 55]. In line with the CONSORT extension for reporting of randomised noninferiority trials [27], noninferiority was considered to be demonstrated if the difference between Adapted PACE and original PACE was no more than the ∆ of 16.4 minutes, based on examination of the 95% credible interval (as per the estimate provided by the posterior probability distribution [56, 57]. As a favourable result in this study’s outcome is an increase in minutes of total physical activity implemented, the noninferiority margin was set as − 16.4 minutes; with noninferiority of Adapted PACE determined if the lower 95% credible interval does not cross the ∆.. The same process informed ∆ for the secondary trial outcomes: − 8.25 minutes for energisers, − 1.58 minutes for active lessons and − 0.95 minutes for PE.

#### Cost-minimisation analysis

The total delivery cost and average cost per school overall and by strategy were calculated for both groups. The between-group difference in costs were calculated for overall total cost and average cost per school. Non-parametric bootstrapping analysis with 1000 replications was used to calculate uncertainty intervals to account for sampling variability.

## Results

### School and participant characteristics

**Fig. 1:**
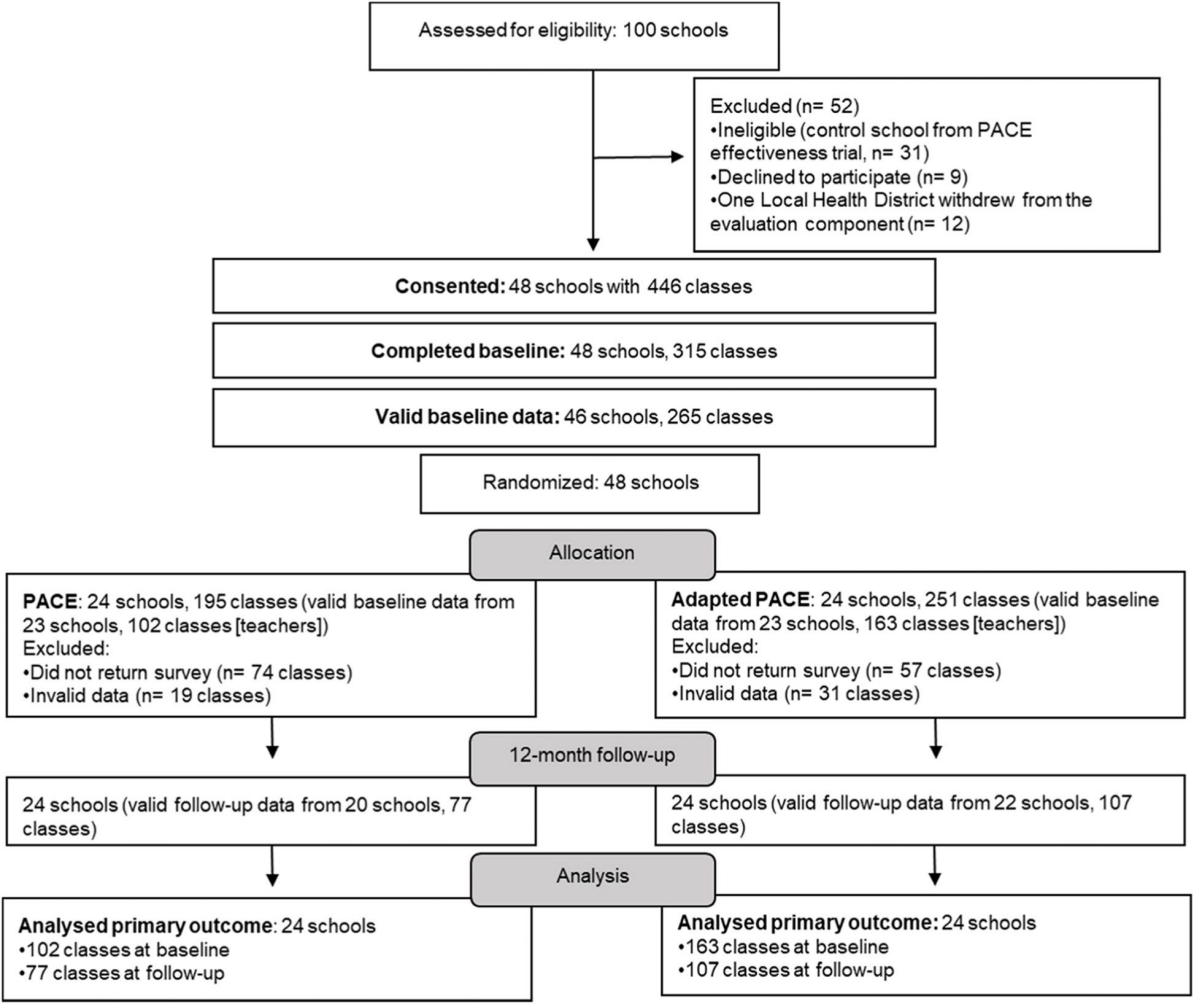
Flowchart of school enrolment and data collection throughout the study

Figure 1 provides a flow diagram of schools and participants through the study. A total of 48 schools with 446 classes were eligible and consented to participate. The characteristics of schools at baseline were similar between groups (Table 2). Of the remaining 48 consenting schools, 6 provided invalid data (i.e., surveys with ≥250 minutes scheduled across 5 days), leaving a total of 42 schools contributing valid data at 12-month follow-up, from a total of 104 teachers. The characteristics of teachers at baseline and follow-up were similar between groups (Table 3).

**Table 2:** School characteristics at baseline by experimental group

School characteristics	PACE *N* = 24	Adapted PACE *N* = 24
School type		
• Catholic	2 (8%)	3 (13%)
• Government	21 (88%)	20 (83%)
• Independent	1 (4%)	1 (4%)
Number of students (size)		
• Mean (SD)	205.9 (199.9)	242 (252.3)
Socio-Economic Indexes for Areas (SEIFA)^a^		
• Most disadvantaged	17 (71%)	16 (67%)
• Least disadvantaged	7 (29%)	8 (33%)
Geolocation		
• Major city	11 (46%)	11 (46%)
• Inner/outer regional or remote	13 (54%)	13 (54%)

^a^SEIFA: relative socio-economic advantage and disadvantage

**Table 3:** Teacher characteristics at baseline and follow-up by experimental group

Teacher variable	PACE		Adapted PACE	
	Baseline	Follow-up	Baseline	Follow-up
School type teaching at	*N = 102*	*N = 77*	*N = 163*	*N = 107*
• Catholic	8 (8%)	7 (9%)	22 (14%)	6 (6%)
• Government	77 (75%)	66 (86%)	141 (87%)	80 (75%)
• Independent	17 (17%)	4 (5%)	0 (0%)	21 (20%)
Age	*N = 77*	*N = 62*	*N = 112*	*N = 94*
• mean (SD)	39.1 (12.0)	38.4 (11.1)	41.8 (11.8)	40.6 (11.1)
Sex	*N = 85*	*N = 71*	*N = 156*	*N = 105*
• Female	76 (89%)	63 (89%)	124 (79%)	82 (78%)
• Male	9 (11%)	8 (11%)	32 (21%)	23 (22%)
Employment status	*N = 85*	*N = 67*	*N = 130*	*N = 105*
• Full-time	77 (91%)	61 (91%)	115 (88%)	92 (88%)
• Part-time/casual	8 (9%)	6 (9%)	15 (12%)	13 (12%)
Years teaching experience	*N = 85*	*N = 67*	*N = 127*	*N = 103*
• mean (SD)	12.8 (9.9)	10.5 (8.9)	15.2 (11.2)	15.8 (11.8)
Specialist PE teacher	*N = 84*	*N = 67*	*N = 132*	*N = 104*
• yes	0 (0%)	1 (1%)	1 (1%)	2 (2%)

### Primary outcome: weekly minutes of structured physical activity implemented by classroom teachers

**Fig. 2:**
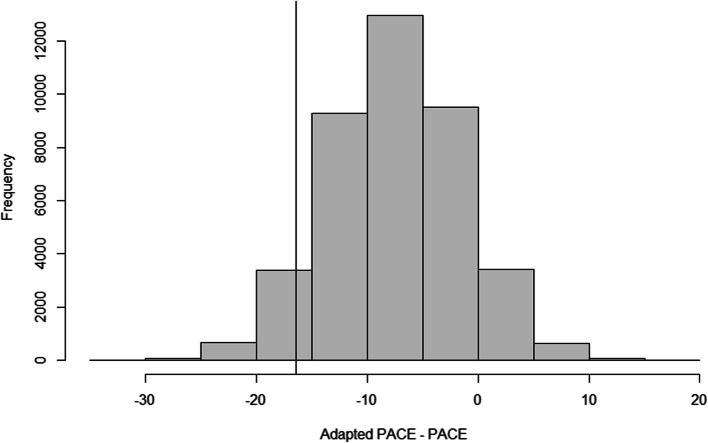
Distribution of the posterior estimated differences in teacher's total scheduled minutes of physical activity between groups (uninformative prior)

Figure 2 displays the distribution of the posterior estimated differences between groups in teacher's total scheduled minutes of physical activity using the uninformative prior. The posterior estimate for the baseline adjusted difference was -7.48 minutes; with a 95% probability that the true difference lies between -19.18 and 4.10 minutes. Only 6.6% of the posterior samples crossed the ∆ of -16.4 minutes, resulting in a 93.4% probability of Adapted PACE being considered noninferior to PACE (i.e., Adapted PACE was no more than 16.4 minutes less than the original model) (see Table 4). The results were identical when the informative prior distribution was used.

**Table 4:** The mean weekly minutes of physical activity implemented by teachers at baseline and 12-month follow-up with intention-to-treat noninferiority analyses results

Total weekly minutes implemented for:	PACE		Adapted PACE		Between group difference from baseline–follow-up			
	Baseline mean (SD) *N = 102*	Follow-up mean (SD) *N = 77*	Baseline mean (SD) *N = 163*	Follow-up mean (SD) *N = 107*	Posterior estimate (95% credible interval)^c^	Pre-specified ∆	Probability of noninferiority^d^	Variance Ratio (95% CI)
All physical activity	122.16 (48.23)	164.62 (44.96)	130.63 (45.43)	159.63 (34.22)	-7.48 (-19.18, 4.10)^a^	−16.4	93.4%	0.17 (-0.17, 0.4)
Energisers	15.93 (25.75)	38.95 (32.22)	21.62 (29.72)	39.07 (28.44)	1.04 (0.78, 1.38)^b^	−8.25	99.6%	0.09 (−0.19, 0.33
Active lessons	9.91 (16.36)	14.99 (19.88)	11.56 (22.41)	16.07 (20.15)	0.99 (0.58, 1.75)^b^	−1.58	56.0%	0.35 (−0.19, 0.74
PE	47.11 (29.55)	61.16 (40.18)	49.33 (32.14)	51.92 (30.60)	0.92 (0.77, 1.12)^b^	−0.95	16.4%	0.25 (− 0.03, 0.48)

^a^Between group difference at follow-up controlling for baseline values of the outcome

^b^Exponentiated coefficient representing the between group difference in the change from baseline to follow-up

^c^PACE is the reference category for all models so negative values for the primary outcome and values < 1 for the secondary outcomes indicate that scheduling of physical activity was, on average, lower in the Adapted PACE group than PACE

^d^Probability that the true difference is < the pre-specified ∆

### Secondary outcomes: weekly minutes of energisers, active lessons and PE implemented by classroom teachers

Table 4 provides the between group differences in the change from baseline to follow-up for the secondary outcomes. The following posterior estimates and 95% credible intervals for the secondary outcomes have been exponentiated to represent a percentage difference in the minutes of scheduled physical activity between groups. Compared with teachers who received the original PACE model, teachers who received Adapted PACE scheduled 4% higher mean minutes of energisers (exponent of posterior estimate = 1.04; 95% credible interval 0.78, 1.38; noninferiority probability = 99.6%), 8% lower mean minutes of PE (exponent of posterior estimate = 0.92; 95% credible interval 0.77, 1.12; noninferiority probability = 16.4%), and 1% lower mean minutes of active lessons (exponent of posterior estimate = 0.99; 95% credible interval 0.58, 1.75; noninferiority probability = 56%).

### Program delivery costs

The cost of the 1–2 hour educational session for staff was unique to the original PACE model, resulting in a cost of $287 per school. Of the strategies received by both groups. The most costly was the full-day training workshop for in-school champions (mean = $484 per school) followed by the ongoing support provided by project officers (mean = $86 per school) and the physical activity equipment pack (mean = $85 per school).

From the health service provider perspective, the total cost to deliver PACE was $25,375 (95% Uncertainty Interval (UI) = $21,499, $29,106), equating to approximately $1057 per school (95% UI = $896, $1213). The total cost to deliver Adapted PACE was $16,421 (95% UI = $13,974, $19,656), equating to approximately $684 (95% UI = $582, $819) per school. Adapted PACE was associated with cost-savings of approximately $8954 (95% UI = $4161, $13,432) in total delivery costs or $373 (95% UI = $173, $560) per school.

## Discussion

As one study in a sequence of optimisation research, we conducted a robust noninferiority trial to explore the potential of an adapted model of PACE with reduced in-person contact. The findings showed a high probability (93.4%) that Adapted PACE was noninferior to the original PACE model in assisting teachers to implement weekly school day physical activity. Simply put, there is a high likelihood that Adapted PACE is “as good as” [26] original PACE in terms of effectiveness. An in-depth exploration of program implementation, provided in the published mixed methods process evaluation of this trial [58], corroborates these findings. In the current study, cost-minimisation showed substantial savings with the adapted model. The adapted, multi-strategy PACE intervention maintained a meaningful effect at a reduced cost; therefore, it is a more attractive option for achieving policy implementation, and one that may be more amenable to implementing at scale. The results are discussed in light of the literature following.

In a frequentist analysis, if the lower 95% confidence limit crosses the ∆ (when a favourable result is reflected as an increase in the outcome), this suggests, as per the CONSORT extension for noninferiority trials [27], that the findings are ‘inconclusive’ and any claim of noninferiority invalid. Importantly, the CONSORT reporting guidelines [27] are for 95% confidence limits (i.e., a frequentist paradigm), whereas we have presented 95% credible intervals from Bayesian analyses which does not correspond to a frequentist 95% confidence limit as they are not probability distributions. Thus, frequentist approaches cannot produce estimates of the probability of noninferiority. Through Bayesian analyses we have estimated this quantity of interest – i.e., the probability estimate for noninferiority which was a decidedly high 96% for scheduled physical activity and an even higher 99.6% for the scheduling of energisers. It is therefore highly probable that Adapted PACE is noninferior to PACE. Together with the substantially reduced cost, we are satisfied that Adapted PACE is a valuable approach that can achieve program objectives. Under a frequentist approach, we would have been unable able to draw any informative conclusions from the null-effects. As an example, a recent study of a public health program for reducing the risk of falls in older adults assessed whether a group-delivered format was noninferior to an individually delivered format [59]. Similar to our study, the upper bound of the two-sided 95% confidence interval crossed the predefined noninferiority margin, indicating inconclusive results [59]. Whilst the research design was robust, and the authors propose that the actual difference may have been close, the use of a Frequentist analytic approach did not enable interpretation of the null findings or meaningful conclusions.

When examining the individual components of physical activity scheduled, energisers were the only component scheduled at a higher rate by the Adapted PACE group compared to the original PACE group, with an almost 100% probability of being noninferior (99.1%). Energisers are short and do not require dedicated space, extensive resources, additional curriculum time or specialised training in physical education to deliver [60]. Consequently, compared to the other secondary outcomes in this trial (active lessons and PE), energisers may represent an opportune strategy that requires minimal external, in-person support to increase the amount of physical activity delivered by teachers across the school week. Energisers were the prominent area of change in previous trials of PACE [16, 18] also sustained at longer-term follow-up [16]. Our findings emphasize the benefit and ease of energisers as the focus for improving scheduled school day physical activity, and suggest that this is possible via Adapted PACE.

Little is known about adaptations made to implementation interventions, including the processes behind these decisions [51]. Using a rigorous, evidence informed and co-created adaptation process, this study demonstrated that the positive impact of PACE was not lost when delivered with reduced in-person support. This is important as in-person delivery typically requires more from agencies aiming to implement health interventions (e.g., transportation, money and time) [22, 61] and may be an impediment to implementation support particularly for remote or rural areas. Adapted PACE may address inequalities in health services by providing a model of support that enables more equitable access. Additionally, the costs of delivering Adapted PACE per school was nearly two thirds of the original model, effectively enabling a 35% increase in the number of schools that could receive implementation support given a fixed health service budget. Such findings are consistent with similar research of distance-delivered health interventions compared with in-person [61, 62] including the previously optimised school-based nutrition policy implementation strategy [23, 24]. Nonetheless, improvements in efficiency of this magnitude are considerable from a health service perspective and may yield substantial improvements in community health if adopted at scale. Specifically, the AUD $373 cost-savings per school represents sizable savings for scale-up to the remaining 400+ primary schools within the service region (>$149,200) or to the 1600+ primary schools across NSW [46] (>$596,800). The findings provide support for the application of mode of delivery adaptations including those in the school setting [20, 42] to support scale-up [63]. Future research should explore the impact of Adapted PACE delivered at scale to expand on the emerging evidence base of adaptations made to scale-up health interventions [20, 42].

A prominent adaptation made for the current study was the use of in-school champions (i.e., an existing teacher at the school – an extant PACE strategy) instead of the health service, to deliver the training to school teachers. Champions are common in school-based health implementation interventions [40, 41, 64, 65] and may be opportune to assume responsibility for peer-education or similar strategies. In a 2006 three-arm cluster RCT, Naylor and colleagues [40] compared the effectiveness of a multi-strategy school physical activity intervention using two delivery approaches with different cost implications: in-school champions and external liaisons. No significant difference was found between the groups in daily physical activity scheduled by teachers, although both showed improvements compared to a no-intervention control group. The current study substantiates this evidence base and adds explicit cost comparisons. The use of an in-school champion to deliver staff training, rather than an external project officer, resulted in cost-savings of approximately $206 per school. In-school champions may assist with physical activity program delivery (particularly for peer-education) at a reduced cost.

This study is the third in a sequence of RCTs undertaken to optimise PACE. Data from preceding trials were used to establish the priors that enabled the calculation of a robust noninferiority margin and efficient Bayesian analytical methods [66]. It also provided the basis to support the selection of adaptations. In this context, the findings of this study provide support for iterative, data-driven and co-ordinated processes in achieving improvement in implementation approaches. Specifically it provides one means of addressing the challenges to the implementation of physical activity policies that have beset schools internationally for over a decade. It is also consistent with achievements in implementation where similar methods have been employed, such as the use of implementation laboratories to improve diabetes management or prescribing behaviour [67]. The broader application of these processes has tremendous potential to support the implementation of other physical activity policies and indeed, other preventive health initiatives in schools and similar settings.

A number of limitations should be considered when interpreting the trial findings. Measurement of outcomes via teacher's self-report in daily logbooks is at risk of social desirability and recall bias, which may lead to teacher's over-estimation of scheduled physical activity. However, as this was a noninferiority trial, and both arms received an active PACE intervention, any data collection limitations are associated with both trial arms and should not obscure the noninferiority analyses. Teacher logbooks have also been use for all evaluations of PACE to-date (originally chosen based on use in other school-based studies [40, 51, 68] and pragmatics [16, 18]), and a consistent outcome measure may, in future, enable the assessment of any ‘scale-up penalty’ [20]. In addition, the generalisability of the findings are limited as the trial was undertaken within one health service region. Future research should explore the impact of Adapted PACE delivered within other contexts. This may be particularly important to improve physical activity policy implementation where it remains an issue in other Australian states [69] and worldwide [3, 4, 8, 10, 11]. Finally, due to time constraints, the result of a real-world health service delivery context, there is no published protocol. Whilst this increases the risk of reporting bias, the main study methods including the outcome measures and data collection procedures are identical to the other PACE trials, which have previously been published [16, 18]. Moreover, the analysis was planned a-priori in consult with independent senior statisticians who have training and expertise in Bayesian methods.

## Conclusions

This study used a unique research design and analytic approach and showed that the use of scalable delivery modalities substantially reduced the cost of PACE without compromising its effectiveness. The study supports the use of mode of delivery adaptations as a strategy to minimise the relative costs of implementing health interventions, without adversely impacting on their effects. The findings should be of particular interest to health and education policy makers and practitioners interested in maximising the benefits to student health from supporting the large scale implementation of physical activity polices in schools.

## Supplementary Information

**Additional file 1.** The stepwise collaborative process used to determine adaptations for PACE.

**Additional file 2.** Completion of the framework for reporting adaptations and modifications to evidence-based implementation strategies (FRAME-IS*).

## Data Availability

The datasets used for the current study are available from the corresponding author upon reasonable request.

## Abbreviations

WHO: World Health Organization
RCT: Randomised controlled trial
PACE: Physically Active Children in Education
NSW: New South Wales
CC: Central Coast
HNE: Hunter New England
BCW: Behaviour Change Wheel
TDF: Theoretical Domains Framework

## References

[1] World Health Organization. Global action plan on physical activity 2018–2030: more active people for a healthier world. Geneva: World Health Organization; 2018. Available from: https://apps.who.int/iris/handle/10665/272722

[2] Stylianou M, Walker JL. An assessment of Australian school physical activity and nutrition policies. Aust N Z J Public Health. 2018;42(1):16–21. 29235711 10.1111/1753-6405.12751

[3] Olstad DL, Campbell EJ, Raine KD, Nykiforuk CI. A multiple case history and systematic review of adoption, diffusion, implementation and impact of provincial daily physical activity policies in Canadian schools. BMC Public Health. 2015;15(1):385. 25885026 10.1186/s12889-015-1669-6 PMC4436021

[4] Carlson JA, Sallis JF, Chriqui JF, Schneider L, McDermid LC, Agron P. State policies about physical activity minutes in physical education or during school. J Sch Health. 2013;83(3):150–6. 23343315 10.1111/josh.12010

[5] Nørager Johansen DL, Neerfeldt Christensen BF, Fester M, Koch B, Lund Kristensen P, Runge Larsen L, et al. Results from Denmark's 2018 report card on physical activity for children and youth. J Phys Act Health. 2018;15(s2):S341–S3. 30475135 10.1123/jpah.2018-0509

[6] Chen P, Wang D, Shen H, Yu L, Gao Q, Mao L, et al. Physical activity and health in Chinese children and adolescents: expert consensus statement (2020). Br J Sports Med. 2020;54(22):1321. 32471813 10.1136/bjsports-2020-102261 PMC7606574

[7] Public Health England. What works in schools and colleges to increase physical activity? London: PHE publications; 2020. Available from: https://www.gov.uk/government/publications/what-works-in-schools-to-increase-physical-activity-briefing

[8] Oxford Research. Bevægelse i skoledagen 2017 (in Danish). Oxford Resarch: Denmark; 2017. Available from: https://skoleidraet.dk/media/6346522/bevaegelse-i-skoledagen-2017.pdf

[9] New South Wales Auditor-General. Physical activity in government primary schools. Department of Education and Communities. Sydney: Audit Office of NSW; 2012. Available from: https://www.audit.nsw.gov.au/sites/default/files/pdf-downloads/2012_Jun_Report_Physical_Activity_in_Government_Primary_Schools.pdf

[10] Canadian Fitness and Lifestyle Research Institute (CFLRI). School policies supporting physical activity and sport. Ottawa: CFLRI; 2016. Available from: https://cflri.ca/bulletin-01-school-policies-supporting-physical-activity-and-sport

[11] Harrington DM, Belton S, Coppinger T, Cullen M, Donnelly A, Dowd K, et al. Results from Ireland's 2014 report card on physical activity in children and youth. J Phys Act Health. 2014;11(s1):S63–S8. 25426916 10.1123/jpah.2014-0166

[12] Hardman K. Physical education in schools: a global perspective. Kinesiology. 2008;40(1):5–28.

[13] Weatherson KA, Gainforth HL, Jung ME. A theoretical analysis of the barriers and facilitators to the implementation of school-based physical activity policies in Canada: a mixed methods scoping review. Implement Sci. 2017;12(1):41. 28347322 10.1186/s13012-017-0570-3 PMC5369225

[14] Mâsse LC, Naiman D, Naylor P-J. From policy to practice: implementation of physical activity and food policies in schools. Int J Behav Nutr Phys Act. 2013;10(1):71. 23731803 10.1186/1479-5868-10-71 PMC3681662

[15] Gilmore T, Donohoe H. Elementary school generalist teachers’ perceived competence to deliver Ontario's daily physical activity program. Loisir et Société/Society and Leisure. 2016;39(1):135–44.

[16] Nathan N, Hall A, McCarthy N, Sutherland R, Wiggers J, Bauman AE, et al. Multi-strategy intervention increases school implementation and maintenance of a mandatory physical activity policy: outcomes of a cluster randomised controlled trial. Br J Sports Med. 2022;56(7):385–93. 34039583 10.1136/bjsports-2020-103764 PMC8938653

[17] Nathan N, Elton B, Babic M, McCarthy N, Sutherland R, Presseau J, et al. Barriers and facilitators to the implementation of physical activity policies in schools: a systematic review. Prev Med. 2018;107:45–53. 29155228 10.1016/j.ypmed.2017.11.012

[18] Nathan N, Sutherland R, Hope K, McCarthy N, Pettett M, Elton B, et al. Implementation of a school physical activity policy improves student physical activity levels: outcomes of a cluster-randomized controlled trial. J Phys Act Health. 2020;17(10):1009–18. 32919383 10.1123/jpah.2019-0595

[19] Milat A, Lee K, Conte K, Grunseit A, Wolfenden L, Van Nassau F, et al. Intervention Scalability Assessment Tool: A decision support tool for health policy makers and implementers. Health Res Policy Syst. 2020;18(1):1. 31900230 10.1186/s12961-019-0494-2 PMC6942323

[20] Lane C, McCrabb S, Nathan N, Naylor P-J, Bauman A, Milat A, et al. How effective are physical activity interventions when they are scaled-up: a systematic review. Int J Behav Nutr Phys Act. 2021;18(1):16. 33482837 10.1186/s12966-021-01080-4 PMC7821550

[21] Wolfenden L, Bolsewicz K, Grady A, McCrabb S, Kingsland M, Wiggers J, et al. Optimisation: defining and exploring a concept to enhance the impact of public health initiatives. Health Res Policy Syst. 2019;17(1):108. 31888666 10.1186/s12961-019-0502-6 PMC6937822

[22] Beall RF, Baskerville N, Golfam M, Saeed S, Little J. Modes of delivery in preventive intervention studies: a rapid review. Eur J Clin Investig. 2014;44(7):688–96. 24828885 10.1111/eci.12279

[23] Reilly KL, Reeves P, Deeming S, Yoong SL, Wolfenden L, Nathan N, et al. Economic analysis of three interventions of different intensity in improving school implementation of a government healthy canteen policy in Australia: costs, incremental and relative cost effectiveness. BMC Public Health. 2018;18(1):378. 29558931 10.1186/s12889-018-5315-y PMC5859495

[24] Reilly KL, Nathan N, Wiggers J, Yoong SL, Wolfenden L. Scale up of a multi-strategic intervention to increase implementation of a school healthy canteen policy: findings of an intervention trial. BMC Public Health. 2018;18(1):1–10. 10.1186/s12889-018-5786-x PMC6042415 29996817

[25] Nathan N, Wiggers J, Bauman AE, Rissel C, Searles A, Reeves P, et al. A cluster randomised controlled trial of an intervention to increase the implementation of school physical activity policies and guidelines: study protocol for the physically active children in education (PACE) study. BMC Public Health. 2019;19(1):170. 30760243 10.1186/s12889-019-6492-z PMC6375171

[26] Flight L, Julious SA. Practical guide to sample size calculations: non-inferiority and equivalence trials. Pharm Stat. 2016;15(1):80–9. 26604186 10.1002/pst.1716

[27] Piaggio G, Elbourne DR, Pocock SJ, Evans SJ, Altman DG, CONSORT Group ft. Reporting of noninferiority and equivalence randomized trials: extension of the CONSORT 2010 statement. JAMA. 2012;308(24):2594–604. 23268518 10.1001/jama.2012.87802

[28] Campbell MK, Piaggio G, Elbourne DR, Altman DG. Consort 2010 statement: extension to cluster randomised trials. BMJ. 2012;345:e5661. 22951546 10.1136/bmj.e5661

[29] Pinnock H, Barwick M, Carpenter CR, Eldridge S, Grandes G, Griffiths CJ, et al. Standards for reporting implementation studies (StaRI) statement. BMJ. 2017;356:i6795. 28264797 10.1136/bmj.i6795 PMC5421438

[30] U.S. Food and Drug Administration (FDA). Non-Inferiority Clinical Trials to Establish Effectiveness: Guidance for Industry. Rockville: FDA; 2016. Available from: https://www.fda.gov/media/78504/download

[31] Angeli F, Verdecchia P, Vaudo G, Masnaghetti S, Reboldi G. Optimal use of the non-inferiority trial design. Pharm Med. 2020;34(3):159–65. 10.1007/s40290-020-00334-z 32277352

[32] European Medicines Agency (EMA). Guideline on the Choice of the Non-Inferiority Margin. London: EMA; 2005. Available from: https://www.ema.europa.eu/en/choice-non-inferiority-margin

[33] New South Wales Education. 2020 NSW government schools by type and SA4 groupings. NSW Education Data Hub; 2021. Available from: https://data.cese.nsw.gov.au/data/dataset

[34] Health Stats NSW. Population by Local Health District (Hunter New England Local Health District and Central Coast Local Health District). 2016. Retrieved from: https://www.healthstats.nsw.gov.au/#/home

[35] New South Wales (NSW) Government. Sport and physical activity policy. In: Department of Education; 2015. Available from: https://education.nsw.gov.au/policy-library/policies/pd-​2002-​0012.

[36] New South Wales Government. Sport and physical activity policy (Ref: PD-2002-0012-V03.0.2). Department of Education: Sydney; 2015. Available from: https://education.nsw.gov.au/policy-library/policies/pd-2002-0012

[37] Michie S, Atkins L, West R. The behaviour change wheel. 1st ed. Great Britain: Silverback Publishing; 2014.

[38] Michie S, Johnston M, Francis J, Hardeman W, Eccles M. From theory to intervention: mapping theoretically derived behavioural determinants to behaviour change techniques. Appl Psychol. 2008;57(4):660–80.

[39] Wolfenden L, Yoong SL, Williams CM, Grimshaw J, Durrheim DN, Gillham K, et al. Embedding researchers in health service organizations improves research translation and health service performance: the Australian Hunter New England Population Health example. J Clin Epidemiol. 2017;85:3–11. 28341367 10.1016/j.jclinepi.2017.03.007

[40] Naylor P-J, Macdonald HM, Zebedee JA, Reed KE, McKay HA. Lessons learned from Action Schools! BC—an ‘active school’ model to promote physical activity in elementary schools. J Sci Med Sport. 2006;9(5):413–23. 16884957 10.1016/j.jsams.2006.06.013

[41] Sutherland RL, Nathan NK, Lubans DR, Cohen K, Davies LJ, Desmet C, et al. An RCT to facilitate implementation of school practices known to increase physical activity. Am J Prev Med. 2017;53(6):818–28. 29051015 10.1016/j.amepre.2017.08.009

[42] McCrabb S, Lane C, Hall A, Milat A, Bauman A, Sutherland R, et al. Scaling-up evidence-based obesity interventions: A systematic review assessing intervention adaptations and effectiveness and quantifying the scale-up penalty. Obes Rev. 2019;20(7):1–19. 30868745 10.1111/obr.12845

[43] Miller CJ, Barnett ML, Baumann AA, Gutner CA, Wiltsey-Stirman S. The FRAME-IS: a framework for documenting modifications to implementation strategies in healthcare. Implement Sci. 2021;16(1):36. 33827716 10.1186/s13012-021-01105-3 PMC8024675

[44] Australian Curriculum Assessment and Reporting Authority (ACARA). National report on schooling in Australia 2017. Sydney: ACARA; 2017. Available from: https://www.acara.edu.au/reporting/national-report-on-schooling-in-australia/national-report-on-schooling-in-australia-2017

[45] Nathan N, Wolfenden L, Bell AC, Wyse R, Morgan PJ, Butler M, et al. Effectiveness of a multi-strategy intervention in increasing the implementation of vegetable and fruit breaks by Australian primary schools: a non-randomised controlled trial. BMC Public Health. 2012;12:651. 22889085 10.1186/1471-2458-12-651 PMC3490882

[46] Bürkner P-C, Jonah G, Weber S, Johnson A, Modrak M, Badr H, et al. Bayesian regression models using Stan. 2.15.0 ed; 2021.

[47] Lüdecke D, Makowski D, Waggoner P, Patil I. Performance: assessment of regression models performance. R package version 0.9.0; 2022.

[48] Althunian TA, de Boer A, Groenwold RH, Klungel OH. Defining the noninferiority margin and analysing noninferiority: an overview. Br J Clin Pharmacol. 2017;83(8):1636–42. 28252213 10.1111/bcp.13280 PMC5510081

[49] Naylor P-J, Macdonald HM, Warburton DE, Reed KE, McKay HA. An active school model to promote physical activity in elementary schools: action schools! BC. Br J Sports Med. 2008;42(5):338–43. 18272538 10.1136/bjsm.2007.042036

[50] Nettlefold L, Naylor P-J, Macdonald HM, McKay HA. Scaling up action schools! BC: how does voltage drop at scale affect student level outcomes? A cluster randomized controlled trial. Int J Environ Res Public Health. 2021;18(10):5182. 34068235 10.3390/ijerph18105182 PMC8153156

[51] Cradock AL, Barrett JL, Carter J, McHugh A, Sproul J, Russo ET, et al. Impact of the Boston active school day policy to promote physical activity among children. Am J Health Promot. 2014;28(suppl 3):S54–64. 24380467 10.4278/ajhp.130430-QUAN-204

[52] Arem H, Moore SC, Patel A, Hartge P. Berrington de Gonzalez A, Visvanathan K, et al. Leisure time physical activity and mortality: a detailed pooled analysis of the dose-response relationship. JAMA Intern Med. 2015;175(6):959–67. 25844730 10.1001/jamainternmed.2015.0533 PMC4451435

[53] Sanders JP, Biddle SJH, Gokal K, Sherar LB, Skrybant M, Parretti HM, et al. ‘Snacktivity™’ to increase physical activity: Time to try something different? Prev Med. 2021;153:106851. 34662595 10.1016/j.ypmed.2021.106851

[54] Greenhalgh T, Robert G, Macfarlane F, Bate P, Kyriakidou O. Diffusion of innovations in service organizations: Systematic review and recommendations. Milbank Q. 2004;82(4):581–629. 15595944 10.1111/j.0887-378X.2004.00325.x PMC2690184

[55] May CR, Johnson M, Finch T. Implementation, context and complexity. Implement Sci. 2016;11(1):141. 27756414 10.1186/s13012-016-0506-3 PMC5069794

[56] Harms C, Lakens D. Making’null effects’ informative: statistical techniques and inferential frameworks. J Clin Transl Res. 2018;3(suppl 2):382. 30873486 PMC6412612

[57] Bittl JA, He Y. Bayesian analysis: a practical approach to interpret clinical trials and create clinical practice guidelines. Circulation. 2017;10(8):e003563. 28798016 10.1161/CIRCOUTCOMES.117.003563 PMC6421843

[58] Lane C, Naylor PJ, Shoesmith A, Wolfenden L, Hall A, Sutherland R, et al. Identifying essential implementation strategies: a mixed methods process evaluation of a multi-strategy policy implementation intervention for schools. Int J Behav Nutr Phys Act. 2022;19(1):1–22. 35413919 10.1186/s12966-022-01281-5 PMC9004180

[59] Jansen C-P, Nerz C, Labudek S, Gottschalk S, Kramer-Gmeiner F, Klenk J, et al. Lifestyle-integrated functional exercise to prevent falls and promote physical activity: results from the LiFE-is-LiFE randomized non-inferiority trial. Int J Behav Nutr Phys Act. 2021;18(1):115. 34479573 10.1186/s12966-021-01190-z PMC8414469

[60] Masini A, Marini S, Gori D, Leoni E, Rochira A, Dallolio L. Evaluation of school-based interventions of active breaks in primary schools: a systematic review and meta-analysis. J Sci Med Sport. 2020;23(4):377–84. 31722840 10.1016/j.jsams.2019.10.008

[61] El Alaoui S, Hedman-Lagerlöf E, Ljótsson B, Lindefors N. Does internet-based cognitive behaviour therapy reduce healthcare costs and resource use in treatment of social anxiety disorder? A cost-minimisation analysis conducted alongside a randomised controlled trial. BMJ Open. 2017;7(9):e017053. 28899892 10.1136/bmjopen-2017-017053 PMC5595196

[62] Luxton DD, Hansen RN, Stanfill K. Mobile app self-care versus in-office care for stress reduction: a cost minimization analysis. J Telemed Telecare. 2014;20(8):431–5. 25316037 10.1177/1357633X14555616

[63] Sutherland R, Campbell E, Nathan N, Wolfenden L, Lubans DR, Morgan PJ, et al. A cluster randomised trial of an intervention to increase the implementation of physical activity practices in secondary schools: study protocol for scaling up the Physical Activity 4 Everyone (PA4E1) program. BMC Public Health. 2019;19(1):1–14. 31272421 10.1186/s12889-019-6965-0 PMC6610944

[64] Wolfenden L, Nathan NK, Sutherland R, Yoong SL, Hodder RK, Wyse RJ, et al. Strategies for enhancing the implementation of school-based policies or practices targeting risk factors for chronic disease. Cochrane Database Syst Rev. 2017;11(11):CD011677. 29185627 10.1002/14651858.CD011677.pub2 PMC6486103

[65] McKay HA, Macdonald HM, Nettlefold L, Masse LC, Day M, Naylor P-J. Action Schools! BC implementation: from efficacy to effectiveness to scale-up. Br J Sports Med. 2015;49(4):210–8. 25312876 10.1136/bjsports-2013-093361

[66] Gamalo-Siebers M, Gao A, Lakshminarayanan M, Liu G, Natanegara F, Railkar R, et al. Bayesian methods for the design and analysis of noninferiority trials. J Biopharm Stat. 2016;26(5):823–41. 26247350 10.1080/10543406.2015.1074920

[67] Grimshaw J, Ivers N, Linklater S, Foy R, Francis JJ, Gude WT, et al. Reinvigorating stagnant science: implementation laboratories and a meta-laboratory to efficiently advance the science of audit and feedback. BMJ Qual Saf. 2019;28(5):416–23. 30852557 10.1136/bmjqs-2018-008355 PMC6559780

[68] Van Nassau F, Singh AS, Cerin E, Salmon J, Van Mechelen W, Brug J, et al. The Dutch Obesity Intervention in Teenagers (DOiT) cluster controlled implementation trial: intervention effects and mediators and moderators of adiposity and energy balance-related behaviours. Int J Behav Nutr Phys Act. 2014;11:158. 25539582 10.1186/s12966-014-0158-0 PMC4304621

[69] Usher W, Anderton A. Giving the teacher a voice: Perceptions regarding the barriers and enablers associated with the implementation of Smart Moves (compulsory physical activity) within primary state schools. Cogent Educ. 2014;1(1):980383.

