# Correction: Optimising a multi‑strategy implementation intervention to improve the delivery of a school physical activity policy at scale: findings from a randomised noninferiority trial

**DOI:** 10.1186/s12966-025-01776-x

**Published:** 2025-08-13

**Authors:** Cassandra Lane, Luke Wolfenden, Alix Hall, Rachel Sutherland, Patti‑Jean Naylor, Chris Oldmeadow, Lucy Leigh, Adam Shoesmith, Adrian Bauman, Nicole McCarthy, Nicole Nathan

**Affiliations:** 1 School of Medicine and Public Health, The University of Newcastle, Newcastle NSW, 1 University Drive, Callaghan, NSW 2308 Australia; 2 Hunter New England Population Health, Hunter New England Area Health Service, Newcastle, NSW Australia; 3 Priority Research Centre for Health Behaviour, The University of Newcastle, Newcastle, NSW Australia; 4 Hunter Medical Research Institute, New Lambton Heights, NSW Australia; 5 School of Exercise Science, Physical and Health Education, University of Victoria, Victoria, BC Canada; 6 School of Public Health, University of Sydney, Sydney, NSW Australia

**Correction: Int J Behav Nutr Phys Act 19, 106 (2022)**

**https://doi.org/10.1186/s12966-022–01345-6**

Following the publication of the original article [1], the authors identified a coding error that affected the analysis of the primary outcome. The error occurred during a data merge step for the wide-format dataset used in the primary analysis. The authors mentioned that the error did not affect the secondary outcomes or other results, as these utilized the data in long format which was not affected by the merge.

The authors have reanalyzed the primary outcome and updated Table 4 and Fig. 2 accordingly. The corrected analysis results in a slightly larger point estimate and a marginally lower probability of noninferiority, but the overall conclusions of the study remain unchanged.

**Incorrect Table** 4**and Fig.** 2

**Table 4:** The mean weekly minutes of physical activity implemented by teachers at baseline and 12-month follow-up with intention-to-treat noninferiority analyses results

Total weekly minutes implemented for:	PACE		Adapted PACE		Between group difference from baseline–follow-up			
	**Baseline mean (SD)** *N* = 102	**Follow-up mean (SD)** *N* = 77	**Baseline mean (SD)** *N* = 163	**Follow-up mean (SD)** *N* = 107	**Posterior estimate (95% credible interval)** ^**c**^	**Pre-specified**∆	**Probability of noninferiority** ^**d**^	**Variance Ratio (95% CI)**
All physical activity	122.16 (48.23)	164.62 (44.96)	130.63 (45.43)	159.63 (34.22)	−2.23 (−18.02, 14.45)^a^	−16.4	96%	0.10 (−0.4, 0.43)
Energisers	15.93 (25.75)	38.95 (32.22)	21.62 (29.72)	39.07 (28.44)	1.04 (0.78, 1.38)^b^	−8.25	99.6%	0.09 (−0.19, 0.33
Active lessons	9.91 (16.36)	14.99 (19.88)	11.56 (22.41)	16.07 (20.15)	0.99 (0.58, 1.75)^b^	−1.58	56.0%	0.35 (−0.19, 0.74
PE	47.11 (29.55)	61.16 (40.18)	49.33 (32.14)	51.92 (30.60)	0.92 (0.77, 1.12)^b^	−0.95	16.4%	0.25 (−0.03, 0.48)

^a^Between group difference at follow-up controlling for baseline values of the outcome

^b^Exponentiated coefficient representing the between group difference in the change from baseline to follow-up

^c^PACE is the reference category for all models so negative values for the primary outcome and values < 1 for the secondary outcomes indicate that scheduling of physical activity was, on average, lower in the Adapted PACE group than PACE

^d^Probability that the true difference is < the pre-specified ∆

**Fig. 2:**
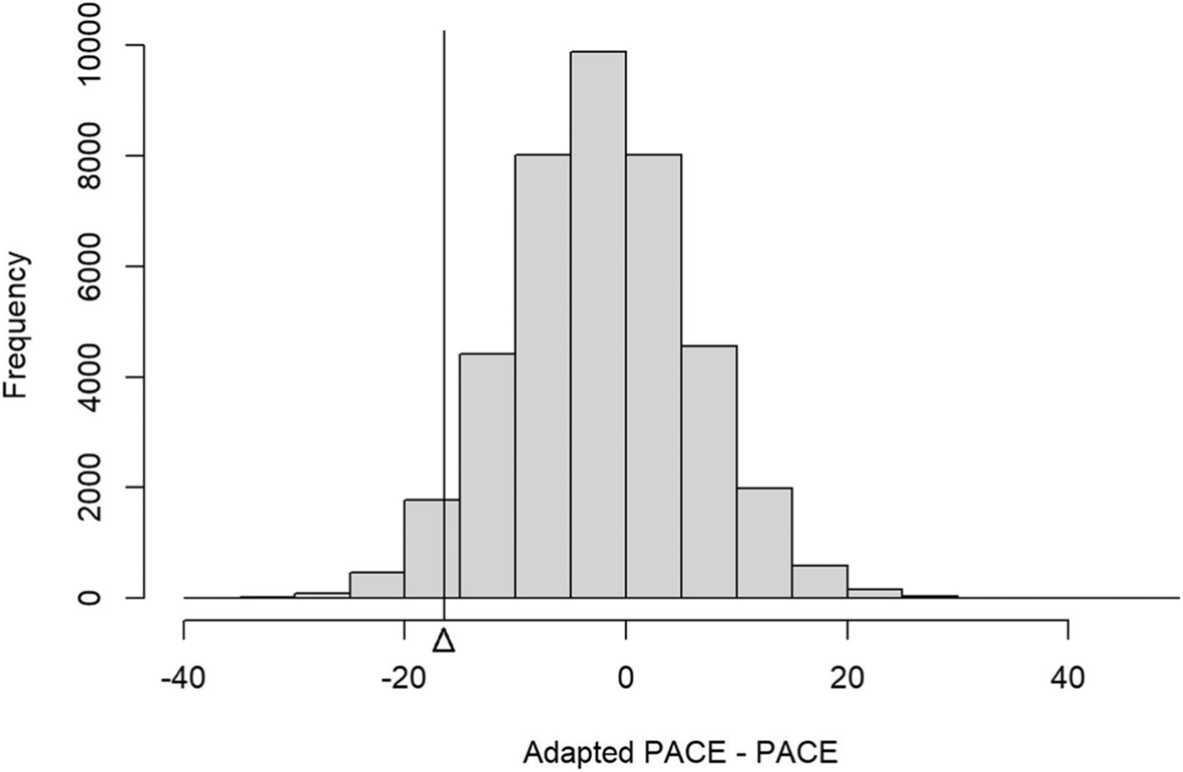
Distribution of the posterior estimated differences in teacher's total scheduled minutes of physical activity between groups (uninformative prior)

**Correct Table** 4** and Fig.** 2

**Table 4:** The mean weekly minutes of physical activity implemented by teachers at baseline and 12-month follow-up with intention-to-treat noninferiority analyses results

Total weekly minutes implemented for:	PACE		Adapted PACE		Between group difference from baseline–follow-up			
	**Baseline mean (SD)** *N* = 102	**Follow-up mean (SD)** *N* = 77	**Baseline mean (SD)** *N* = 163	**Follow-up mean (SD)** *N* = 107	**Posterior estimate (95% credible interval)**^**c**^	**Pre-specified** ∆	**Probability of noninferiority**^**d**^	**Variance Ratio (95% CI)**
All physical activity	122.16 (48.23)	164.62 (44.96)	130.63 (45.43)	159.63 (34.22)	−7.48 (−19.18, 4.10)^a^	−16.4	93.4%	0.17 (−0.17, 0.4)
Energisers	15.93 (25.75)	38.95 (32.22)	21.62 (29.72)	39.07 (28.44)	1.04 (0.78, 1.38)^b^	−8.25	99.6%	0.09 (−0.19, 0.33)
Active lessons	9.91 (16.36)	14.99 (19.88)	11.56 (22.41)	16.07 (20.15)	0.99 (0.58, 1.75)^b^	−1.58	56.0%	0.35 (−0.19, 0.74)
PE	47.11 (29.55)	61.16 (40.18)	49.33 (32.14)	51.92 (30.60)	0.92 (0.77, 1.12)^b^	−0.95	16.4%	0.25 (−0.03, 0.48)

^a^Between group difference at follow-up controlling for baseline values of the outcome

^b^Exponentiated coefficient representing the between group difference in the change from baseline to follow-up

^c^PACE is the reference category for all models so negative values for the primary outcome and values < 1 for the secondary outcomes indicate that scheduling of physical activity was, on average, lower in the Adapted PACE group than PACE

^d^Probability that the true difference is < the pre-specified ∆

**Fig. 2:**
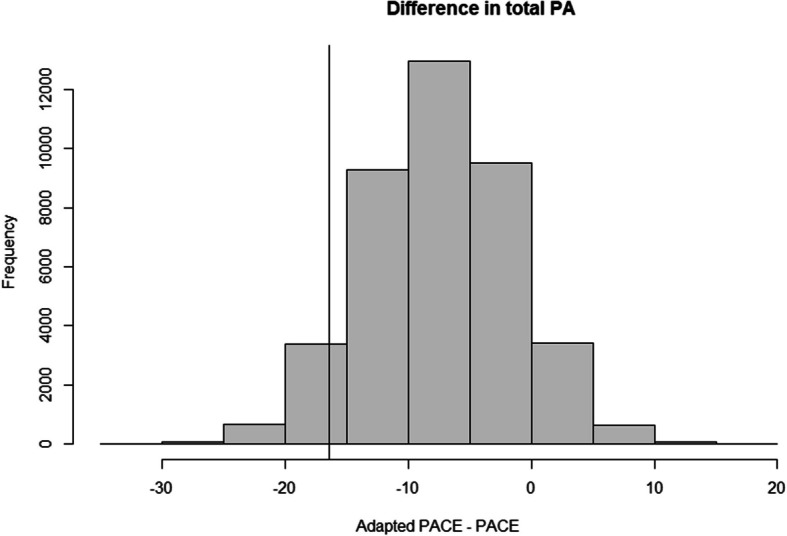
Distribution of the posterior estimated differences in teacher's total scheduled minutes of physical activity between groups (uninformative prior)

Some texts have also been corrected:

**Incorrect Abstract Results**

The posterior estimate for the between group difference at follow-up was − 2.3 min (95% credible interval =  − 18.02, 14.45 min). There was an estimated 96% probability of Adapted PACE being considered noninferior (only 4% of the posterior samples crossed the noninferiority margin of − 16.4 minutes).

**Correct Abstract Results**

The posterior estimate for the between group difference at follow-up was −7.48 min (95% credible interval = −19.18, 4.10 min). There was an estimated 93.4% probability of Adapted PACE being considered noninferior (only 6.6% of the posterior samples crossed the noninferiority margin of −16.4 minutes).

**Incorrect Results**

The posterior estimate for the baseline adjusted difference was − 2.23 min; with a 95% probability that the true difference lies between − 18.02 and 14.45 min. Only 4% of the posterior samples crossed the Δ of − 16.4 min, resulting in a 96% probability of Adapted PACE being considered noninferior to PACE (i.e., Adapted PACE was no more than 16.4 min less than the original model) (see Table 4).

**Correct Results**

The posterior estimate for the baseline adjusted difference was −7.48 min; with a 95% probability that the true difference lies between −19.18 and 4.10 min. Only 6.6% of the posterior samples crossed the ∆ of −16.4 min, resulting in a 93.4% probability of Adapted PACE being considered noninferior to PACE (i.e., Adapted PACE was no more than 16.4 min less than the original model) (see Table 4).

**Incorrect Discussion**

The findings showed a high probability (96%) that Adapted PACE was noninferior to the original PACE model in assisting teachers to implement weekly school day physical activity.

**Correct Discussion**

The findings showed a high probability (93.4%) that Adapted PACE was noninferior to the original PACE model in assisting teachers to implement weekly school day physical activity.

The original article has been corrected.
