# Supplementary material for: Optimising a multi-strategy implementation intervention to improve the delivery of a school physical activity policy at scale: findings from a randomised noninferiority trial
Source: Int J Behav Nutr Phys Act. 2022 Aug 20;19:106. doi: 10.1186/s12966-022-01345-6 (PMC9392334; doi:10.1186/s12966-022-01345-6)
Supplement: Supplementary file 1 — Additional file 1. The stepwise collaborative process used to determine adaptations for PACE. [file 12966_2022_1345_MOESM1_ESM.docx]

**Additional file 1**: The stepwise collaborative process used to determine adaptations for PACE

The adaptation process was guided by the Collaborative Intervention Planning Framework (CIPF) – an integration of community based participatory research (CBPR) principles with intervention mapping (IM) procedures (1). An emphasis was placed on shared decision making between researchers, the local health service and school stakeholders to enhance the implementation potential of the resultant intervention (2). The following steps were taken to determine adaptations:

1. Each strategy was positioned within a decision making matrix (see below) based on perceived effectiveness and average cost. Strategy placement was informed by (i) surveys of health service staff experienced in supporting schools to implement the physical activity policy, and implementation scientists involved in the conduct of the original PACE research trials. The survey required strategies to be ranked from 1=most effective to 8=least effective, (ii) calculations of the cost to deliver each strategy using PACE trial records.
2. A literature review of delivery modalities used in public health, implementation research and/or physical activity interventions was conducted. This provided evidence suggesting that a range of delivery modalities may be as effective in achieving behavioural change as in-person delivery. These alternate mode also had the potential to increase reach, and efficacy (3,4,5).
3. A multidisciplinary workshop of researchers, health practitioners, project officers and school stakeholders was convened to explore possible mode of delivery adaptations based on the findings of the surveys (step 1) and literature reviews (step 2). We prioritised strategies categorised as both ‘highly effective’ and ‘high cost’ based on surveys of health service staff and implementation scientists described in step 1.
4. An evidence-informed, adapted model of PACE which met the needs of stakeholders and fit within resource constraints of the health service organisation was proposed. Table 4.1 within the manuscript includes an overview of the mode of delivery adaptations that were made to several PACE implementation strategies. Additional file 2 provides a report of the adaptations in accordance with the Framework for Reporting Adaptations and Modifications to Evidence-based Implementation Strategies (FRAME-IS) (6).

Low Effective

Low Cost

Low Effective

High Cost

Highly Effective

High Cost

Highly Effective

Low Cost

**Cost**

**Perceived Effectiveness**

PACE Implementation Strategies:

1. Centralise technical assistance and provide ongoing consultation
2. Mandate change
3. Identify and prepare in-school champions
4. Develop a formal implementation blueprint
5. Conduct educational outreach visits
6. Develop and distribute educational materials
7. Capture and share local knowledge
8. Change physical structure and equipment

**1**

**2**

**3**

**4**

**5**

**6**

**7**

**8**

**References**

1. Cabassa LJ, Gomes AP, Meyreles Q, Capitelli L, Younge R, Dragatsi D, et al. Using the collaborative intervention planning framework to adapt a health-care manager intervention to a new population and provider group to improve the health of people with serious mental illness. Implementation Science. 2014;9(1).
2. Durlak JA, Dupre EP. Implementation Matters: A Review of Research on the Influence of Implementation on Program Outcomes and the Factors Affecting Implementation. American Journal of Community Psychology. 2008;41(3-4):327-50.
3. Beall RF, Baskerville N, Golfam M, Saeed S, Little J. Modes of delivery in preventive intervention studies: a rapid review. European Journal of Clinical Investigation. 2014;44(7):688-96.
4. Castro CM, Pruitt LA, Buman MP, King AC. Physical activity program delivery by professionals versus volunteers: the TEAM randomized trial. Health Psychology. 2011;30(3):285.
5. Ginis KAM, Nigg CR, Smith AL. Peer-delivered physical activity interventions: an overlooked opportunity for physical activity promotion. Translational behavioral medicine. 2013;3(4):434-43.
6. Miller CJ, Barnett ML, Baumann AA, Gutner CA, Wiltsey-Stirman S. The FRAME-IS: a framework for documenting modifications to implementation strategies in healthcare. Implement Science. 2021;16(1):36.
