# Supplementary material for: Optimising a multi-strategy implementation intervention to improve the delivery of a school physical activity policy at scale: findings from a randomised noninferiority trial
Source: Int J Behav Nutr Phys Act. 2022 Aug 20;19:106. doi: 10.1186/s12966-022-01345-6 (PMC9392334; doi:10.1186/s12966-022-01345-6)
Supplement: Supplementary file 2 — Additional file 2. Completion of the framework for reporting adaptations and modifications to evidence-based implementation strategies (FRAME-IS*). [file 12966_2022_1345_MOESM2_ESM.docx]

**Additional file 2**: Completion of the framework for reporting adaptations and modifications to evidence-based implementation strategies (FRAME-IS*)

| FRAME-IS module or sub-component | Details |
| --- | --- |
| Module 1 | |
| The EBP being implemented is: | The NSW Department of Education Sport and Physical Activity Policy |
| The implementation strategy being modified is: | Physically Active Children in Education (PACE) |
| The modification(s) being made is/are: | -Strategy 1: Centralised technical assistance and provision of ongoing consultation delivered via email/telephone rather than in-person  -Strategy 2a: Mandate change; initial meeting with school principal delivered via email/telephone rather than in-person  -Strategy 5: Educational outreach visits delivered by an in-school champion rather than an external project officer |
| The reason(s) for the modification(s) is/are: | -reduce in-person contact (to improve scalability of PACE) |
| Module 2 |  |
| What is modified? | -Content (details provided in Module 3)  -Context (personnel; PACE implementation strategy 5 [educational outreach] delivered by an in-school champion rather than an external project officer) |
| Module 3 | |
| What is the nature of the content, evaluation, or training modification? | Tailoring (specifically, mode of delivery for strategy 1 and strategy 2a modified to distance communication rather than in-person) |
| OPTIONAL: what is the relationship to core elements? | Fidelity consistent/Core elements or functions preserved |
| Module 4 | |
| What is the goal? | Improve scalability of the implementation strategy (PACE) as part of an optimisation process |
| What is the level of the rationale for the modification? | - Organisational level (address accessibility and resource issues faced by remote/rural schools and/or those in low SES regions)  - Implementer level (address the limited resources available by public health providers) |
| Module 5 | |
| When is the modification initiated? | Implementation phase |
| Is the modification planned? | Planned/proactive |
| Module 6 | |
| Who participates in the decision to modify? | Program manager, implementation strategy experts (PACE project officers), researchers, and recipients who are the ultimate target of the EBP being implemented (school stakeholders) |
| Module 7 | |
| How widespread is the modification? | -Group of recipients for whom the EBP is being implemented (specifically the 24 schools allocated to Adapted PACE)  -Implementation/facilitation team (the team responsible for implementing PACE) |

* Miller CJ, Barnett ML, Baumann AA, Gutner CA, Wiltsey-Stirman S. The FRAME-IS: a framework for documenting modifications to implementation strategies in healthcare. Implement Science. 2021;16(1):36.
